# Bioluminescence ATP Monitoring for the Routine Assessment of Food Contact Surface Cleanliness in a University Canteen

**DOI:** 10.3390/ijerph111010824

**Published:** 2014-10-17

**Authors:** Andrea Osimani, Cristiana Garofalo, Francesca Clementi, Stefano Tavoletti, Lucia Aquilanti

**Affiliations:** Department of Agricultural, Food and Environmental Sciences, Polytechnic University of Marche, via Brecce Bianche, Ancona 60131, Italy; E-Mails: a.osimani@univpm.it (A.O.); c.garofalo@univpm.it (C.G.); f.clementi@univpm.it (F.C.); s.tavoletti@univpm.it (S.T.)

**Keywords:** Clean-Trace ATP system, surface cleanliness, HACCP, cleaning control points, hygiene, food safety

## Abstract

ATP bioluminescence monitoring and traditional microbiological analyses (viable counting of total mesophilic aerobes, coliforms and *Escherichia coli*) were used to evaluate the effectiveness of Sanitation Standard Operating Procedures (SSOP) at a university canteen which uses a HACCP-based approach. To that end, 10 cleaning control points (CPs), including food contact surfaces at risk of contamination from product residues or microbial growth, were analysed during an 8-month monitoring period. Arbitrary acceptability limits were set for both microbial loads and ATP bioluminescence readings. A highly significant correlation (*r* = 0.99) between the means of ATP bioluminescence readings and the viable counts of total mesophilic aerobes was seen, thus revealing a strong association of these parameters with the level of surface contamination. Among CPs, the raw meat and multi-purpose chopping boards showed the highest criticalities. Although ATP bioluminescence technology cannot substitute traditional microbiological analyses for the determination of microbial load on food contact surfaces, it has proved to be a powerful tool for the real time monitoring of surface cleanliness at mass catering plants, for verify the correct application of SSOP, and hence for their implementation/revision in the case of poor hygiene.

## 1. Introduction

Food-borne diseases are a public health priority since each year a large number of people become ill owing to the consumption of unsafe food; the main concerns are usually related to the presence of physical and chemical contaminants as well as pathogenic microorganisms [1,2]. Microbiological contamination of foods can be ascribed to naturally contaminated raw materials [3] or cross-contamination events, the latter generally caused by microorganisms originating from various sources, namely air, water, human or animal faeces, mucus, hair, infected wounds, dirt, dust, *etc.* [4,5,6] A high standard of hygiene in the work environment (surfaces, equipment, and utensils) is a fundamental requisite for the prevention of microbial contaminations, and hence for obtaining safe foods. Several pathogens, including *Staphylococcus aureus*, *Listeria monocytogenes*, *Salmonella* spp., *Campylobacter jejuni*, *Yersinia enterocolitica* and enteropathogenic strains of *Escherichia coli* can survive on different surfaces for periods ranging from several hours to days [7,8,9] and even form biofilms. The latter are surface-associated microbial communities, consisting of micro-colonies entrapped in an exopolymeric matrix [10], where microbial cells can persist and survive decontamination procedures, thereby representing a potential reservoir for food contamination. In food production plants, the formation of biofilms generally starts when cleaning and sanitation procedures are not performed correctly, and the food residues that remain on the not properly cleaned surfaces constitute a source of nutrients for the microorganisms which may be present [11].

Currently, food safety is guaranteed through the application of the Hazard Analysis Critical Control Point (HACCP) system, which was made mandatory by Regulation EC 852/2004 [12] promulgated by the European Union in order to protect consumers against potential health risks. The HACCP system basically relies on the application of codified procedures, including Sanitation Standard Operating Procedures (SSOPs); the latter include a series of very concise documented instructions that must be followed to ensure an adequate level of hygiene of the surfaces intended for contact with food [13,14]. All HACCP plans specify that the SSOPs are subject to annual and/or supplementary review, the latter whenever there are any modifications to the food production procedures. The SSOPs must be performed by qualified and trained staff; the evaluation of a SSOP is carried out after surface cleaning and sanitation, generally through direct visual inspection and microbiological analyses. The former are rapid and without cost, but are very subjective [15], whereas microbiological tests, mainly relying on surface sampling with contact plates or swabs and further viable cell counting, are internationally accepted, but laborious and time-consuming [16].

In recent decades, alternative more rapid methods have been developed for the real-time evaluation of the cleanliness of food contact surfaces. One of these methods relies on the measurement of the bioluminescence produced by the firefly (*Photinus pyralis*) luciferase through the oxidative decarboxylation of luciferin in the presence of adenosine triphosphate (ATP), a molecule occurring in either living organisms or food, as non-microbial ATP [16]. The amount of light emitted, measured with a luminometer, which consists of a photomultiplier and an amplifier connected to a recorder [16], is strictly dependent on both surface abiotic and biotic contamination; it is expressed as relative light units (RLU).

This study aimed to investigate the introduction of ATP bioluminescence measurement as a real-time and routine verification of the cleaning procedures applied at a university canteen that produces up to 1,200 meals a day using a HACCP-based approach. To that end, data from luminometric tests and surface hygiene swabbing coupled with viable counting of total mesophilic aerobes, coliforms and *Escherichia coli* were collected during an 8-month monitoring period from 10 selected food contact surfaces at risk of contamination from food residues or microbial growth.

## 2. Experimental Section

### 2.1. Description of the Facility

The university canteen taking part in the study has previously been described by Osimani *et al*. [5]. When operating at full capacity, it produces up to 1,200 meals a day; this facility consists of a first floor, comprising the kitchen, the food distribution area and the adjacent dining area, and of a ground floor, divided into separate areas for the handling of raw materials. The HACCP system was adopted by the university canteen in 1997, in accordance with the Directive 93/43/ECC (later replaced with EC Regulation 852/2004 [12]) and was revised in 2004 and 2007. In accordance with the Deliberation of the Marche Region n. 2173 ME/SAN of 10/12/2002 [17], since 2003 the canteen staff has been involved in two training sessions per year, focusing on specific key issues related to food hygiene (e.g., application of effective cleaning and sanitation procedures, proper food storage, *etc.*).

### 2.2. Sampling

The degree of cleanliness of 10 selected food contact surfaces (cleaning control points) at risk of contamination from food residues or microbial growth (Table 1) was assessed during an 8-month monitoring period, spanning from June 2013 to January 2014. Control points (CPs) consisted in easy-to-clean direct contact surfaces at high contamination risk, situated in the following canteen areas: raw meat preparation area, raw vegetable preparation area, and meal distribution area; some of these CPs (raw meat and multipurpose chopping-boards, slicing machine, raw meat knife, raw meat table, and raw vegetable table) corresponded to those already established within the sanitation and manteinance prerequisite programme.

Each surface was sampled 40 times in 40 visits regularly distributed throughout the 8-month monitoring period; for each surface, two adjacent 100 cm^2^ areas were sampled and subjected to ATP bioluminescence measurements and traditional hygiene swabbing (microbiological analyses), respectively. For customer knives, characterised by a food contact surface lower than 100 cm^2^, sampling was carried out on additional analogous surfaces until 100 cm^2^ of total swabbed area was achieved.

**Table 1:** Relative light units (RLU)/100 cm^2^ measured on each surface before and immediately after cleaning and sanitation operations for preliminary definition of ATP bioluminescence acceptability limits.

Class	Control Point (Food Contact Surface)		RLU/100 cm^2^										
			before Routine Cleaning				after Routine Cleaning						Acceptability Limit
			N	Min	Max	Mean	N	Min	Max	Mean	Reduction %	Mean Range	
1	Vegetable washer	Stainless steel	10	755	91,047	13,340.9	10	7	121	36.5	99.7	<60	<100
	Canteen table	PVC	10	312	1,700	768.4	10	18	91	53.2	93.0		
2	Raw meat preparation table	Stainless steel	10	10,529	677,979	166,071.2	10	43	282	100.9	99.9	>60 <150	<150
	Raw meat knife	Stainless steel	10	10,372	158,085	41,434.3	10	37	115	61.9	99.8		
	Raw vegetable preparation table	Stainless steel	10	3,866	217,423	66,649.5	10	33	453	141.6	99.7		
	Raw vegetable knife	Stainless steel	10	7,215	531,768	268,003.4	10	23	904	156.2	99.9		
	Cooked meat slicing machine	Stainless steel	10	1,059	422,253	196,957.9	10	36	200	104.1	99.9		
	Knife for customers	Stainless steel	10	9,304	206,510	61,014.5	10	20	162	64.8	99.8		
3	Raw meat chopping-board	Nylon	10	5,519	208,112	57,858.8	10	56	810	368.3	99.3	>150	<400
	Multipurpose chopping-board	Nylon	10	1,983	189,785	47,986.2	10	24	798	220.3	99.5		

Note: N: Number of replicates.

### 2.3. Cleaning Procedures

During the monitoring period, routine cleaning and sanitation of CPs was carried out in accordance with the HACCP manual; briefly, customer knives were first immersed in a 2% benzalkonium chloride-based solution for approximately 10 min, and further washed in an automatic dishwasher using hot water (50 °C) and an anionic surfactant solution; the remaining CPs were cleaned of coarse dirt (e.g., food residues, packaging waste, *etc.*) using kitchen paper, treated with an anionic surfactant solution, and further subjected to sanitation with a 2% benzalkonium chloride-based solution, following the manufacturer’s instructions for contact times. Once a week (usually on Friday afternoon, after the serving of the last meal), work surfaces and tools were subjected to vigorous sanitation using a 5% sodium hypochlorite solution.

### 2.4. Luminometric Analyses

ATP bioluminescence measurements were performed using the Clean-Trace ATP surface test (UXL100 ATP Test swabs, 3M Health Care, Bracknell, UK) and the bioluminescence reader Clean-Trace NG Luminometer (3M), supplied with a data trending software (Data Trending Software, 3M) allowing filtering, sorting, charting and graphing of data. Measurements were carried out by a trained data collector after either vigorous (for definition of acceptability limits) or routine (for CP monitoring) cleaning and sanitation. The absence of any interference of the sanitiser (2% benzalkonium chloride-based solution) with the ATP bioluminescence reaction was assumed, based on the indications of the bioluminometer supplier. The results of the ATP measurements were expressed as Relative Light Units (RLU)/100 cm^2^.

### 2.5. Microbiological Analyses

Traditional hygiene swabbing was performed on areas adjacent (100 cm^2^) to those subjected to bioluminescence measurements. Microbiological samples were collected using sterile cotton swabs and tubes containing 10 mL of sterile 0.1% peptone solution (Oxoid, Basingstoke, UK) added with 30 g/L of Tween 80 (Liofilchem, Roseto, Italy) for the inactivation of any possible residues of the disinfecting agent used for sanitation. Samples were transferred to the laboratory under refrigerated conditions and immediately subjected to viable cell counting; in more detail, samples were serially ten-fold diluted in a sterile peptone-saline solution (1 g/L peptone and 8.5 g/L NaCl) and aliquots (1 mL) of each dilution were used for counting the following microorganisms: total mesophilic aerobes on Petrifilm AC (3M, St. Paul, MN, USA) incubated at 30 °C for 48 h; coliforms and *Escherichia coli* on Petrifilm EC (3M), incubated at 37 °C for 24 h. The inoculum (1 mL) was spread on the plates by lifting the top layer in order to expose the Petrifilm plating surface; the top film was then slowly rolled down and the “3M spreader” was used for even distribution. Bacterial cell counts were expressed as colony forming units (cfu)/100 cm^2^.

### 2.6. Definition of Luminometric and Microbiological Acceptability Limits

Acceptability limits based on ATP bioluminescence were defined through a series of preliminary analyses carried out on the same surfaces (CP) subjected to routine analyses. In more detail, for each CP, reference values for the maximum levels of dirt and cleanliness were defined by measuring RLU values before and immediately after vigorous cleaning and sanitation, respectively; hence, 20 measurements at each surface, carried out before (10 measurements) and after vigorous cleaning and sanitation (10 measurements) were taken over the course of 10 days using the Clean-Trace ATP surface test (3M) and the bioluminescence reader Clean-Trace NG Luminometer (3M); at the end of this step, the appropriateness of the cleaning and sanitation procedures was verified through the calculation of RLU percentage reduction before and after cleaning.

For the microbiological viable counts, acceptability limits were established on the basis of international guidelines, as previously reported by Osimani and colleagues [18]. In more detail, bacteriological thresholds where chosen in line with the microbiological criteria adopted in the Commission Decision 2001/471/EC laying down rules for the regular checks on the general hygiene carried out by operators in establishments according to Directive 64/433/EEC on health conditions for the production and marketing of fresh meat and Directive 71/118/EEC on health problems affecting the production and placing on the market of fresh poultry meat [19]. Hence, viable counts of coliforms and *E. coli* > 1 CFU/cm^2^ and of total mesophilic aerobes > 10 CFU/cm^2^ were considered as unacceptable, irrespective of the CP.

### 2.7. Statistical Analyses

Both RLU values and viable counts of total mesophilic aerobes and coliforms assessed after routine cleaning were subjected to Two-Way Analysis of Variance (ANOVA) considering surfaces and sampling time as main effects and their interaction as the error term [20] (Statistica, StatSoft, Tulsa, OK, USA); the ANOVA model was: *y_ij_* = µ + α*_i_ +* β*_j_+* Ɛ*_ij_*, where µ is the overall mean, *α_i_* is the effect of the *i*th level of factor “surface” (*i* = 1,…,10), *β_j_* is the effect of the *j*th level of factor “time” (*j* = 1,…,40), and Ɛ*_ij_* is the random error. Multiple comparisons among means were conducted by the Honest Significant Difference (HSD) test. Simple correlation was evaluated by the Pearson correlation coefficient calculated using variable means of each surface.

## 3. Results and Discussion

### 3.1. Definition of ATP Bioluminescence Acceptability Limits

The appropriateness of the cleaning and sanitation procedures used was first verified through preliminary ATP bioluminescence measurements aimed at defining the reference RLU values for the maximum levels of dirt and cleanliness, respectively. In more detail, an RLU percentage reduction >99.3% was seen in almost all the CPs, except for the canteen table, which showed a slightly lower but still appreciable reduction (Table 1), thus suggesting the efficacy of the Sanitation Standard Operating Procedures (SSOP) adopted.

As expected, the different CPs analysed had quite different levels of risk, and therefore required different acceptability thresholds. In more detail, different critical issues were assumed on the basis of differences in the type of materials composing the CPs and the intended use of the latter. As concerns nylon chopping boards, characterized by porous surface, higher reference limits are justified by the formation of superficial cuts and cracks, which render the cleaning difficult. Stainless steel surfaces are easier to clean and therefore, more stringent limits can be established for CP made of this material. Analogously, the vegetable washer and the canteen tables can be assumed to be at low risk; the first is made of stainless steel and it is used with large quantities of water and chlorine, thus implying a high expected sanitization level, and in turn, the establishment of more restrictive acceptability thresholds. Canteen tables, made of smooth PVC, are generally easy to clean, and not subject to wear. In more detail, three classes of routinely cleaned surfaces were arbitrarily defined on the basis of mean RLU/100 cm^2^: <60 (class 1), comprised between 60 and 150 (class 2), and >150 (class 3), respectively; acceptability limits (benchmark values) <100, <150 and <400 RLU/100 cm^2^ were hence established for classes 1, 2 and 3, respectively (Table 1).

### 3.2. Luminometric Analyses after Routine Cleaning and Sanitation

The percentages of non-compliance based on the ATP bioluminescence tests are shown in Table 2.

**Table 2:** Frequency of non-conforming samples based on RLU and bacterial viable counts (TMA, C and Ec) after routine cleaning and sanitation of food contact surfaces.

Surface	N	RLU	TMA	C	Ec
Raw meat preparation table	40	42.5%	7.5%	0%	n.d.
Raw meat chopping-board	40	77.5%	47.5%	20.0%	n.d.
Raw meat knife	40	67.5%	10.0%	5.0%	n.d.
Raw vegetable preparation table	40	72.5%	10.0%	2.5%	n.d.
Raw vegetable knife	40	90.0%	20.0%	2.5%	n.d.
Vegetable washer	40	27.5%	7.5%	0%	n.d.
Multipurpose chopping-board	40	75.0%	52.5%	12.5%	n.d.
Cooked meat slicing machine	40	50.0%	7.5%	0%	n.d.
Table for customers	40	43.3%	0%	0%	n.d.
Knife for customers	40	47.5%	0%	0%	n.d.

Notes: N Number of samples analysed; RLU Relative Light Units; TMA Total Mesophilic Aerobes; C Coliforms; Ec *Escherichia coli*; n.d. never detected.

**Figure 1:**
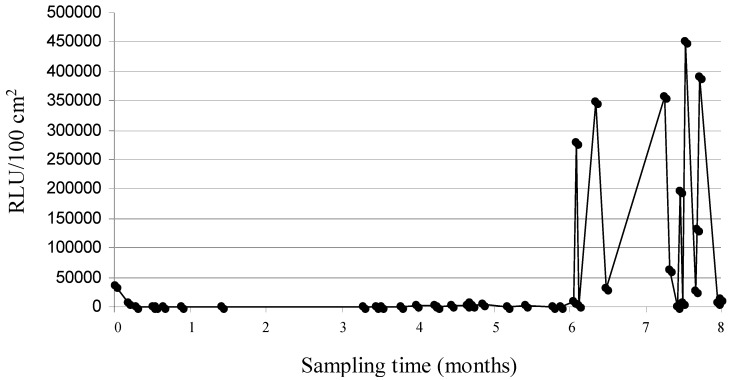
Eight-month RLU measurements trend for raw meat chopping board RLU Relative Light Units.

For most of the food contact surfaces under study, more than 50% of the samples showed not acceptable values of RLU/100 cm^2^, with the raw vegetable knife being characterised by the highest percentage of non-conforming samples (90%). By contrast, a lower percentage of unacceptable samples was found for the vegetable washer (27.5%). The results of ANOVA carried out for the RLU measurements, total mesophilic aerobes (TMA) and coliform counts are shown in Table 3. A remarkable variability between maximum and minimum RLU/100 cm^2^ assessed after routine cleaning and sanitation emerges by evaluating the raw data shown in Table 3. An equally high variability between maximum and minimum RLU/100 cm^2^ was found by Aycicek *et al.* [21] by analysing 14 different cleaned surfaces at a hospital kitchen with the same analytical approach. In our study, this variability was particularly noticeable for the raw meat chopping-board, and to a lesser extent, for the multi-purpose chopping board used for cutting herbs, cheeses, sausages, cooked ham, olives (*etc.*); both these utensils were made of nylon, a porous synthetic material particularly subject to damage during prolonged use, and hence to the formation of cuts and cracks, which render proper cleaning and sanitation particularly troublesome. Regarding the raw meat chopping board, Figure 1 shows the trend in RLU/100 cm^2^ values assessed during the 8-month monitoring period; as clearly emerges from this figure, in the final two months a discontinuous increase in the measured ATP bioluminescence can be noticed, thus supporting the hypothesis of the progressive wear of this utensil. Although raw vegetable and raw meat chopping-boards were made of the same material, the latter was subjected to significantly higher wearing because of the vigorous chopping of bones and cartilaginous tissues, which in turn leads to the formation of deeper cracks and fissures.

Unexpectedly, most of the stainless steel CPs showed high maximum RLU/100 cm^2^ values (Table 3); since stainless steel is universally considered to be particularly suitable for tools, utensils and other food contact surfaces that need routine cleaning and sanitation, the occasional high ATP bioluminescence measured probably suggests isolated cases of inappropriate or hasty application of the SSOP by the canteen staff. A similar conclusion was drawn by Aycicek *et al.* [21], using the same analytical approach to determine surface cleanliness in a hospital kitchen; in both studies, ATP bioluminescence measurements proved to be of particular importance for hygienic kitchen management, especially within HACCP plans.

### 3.3. Microbiological Analyses after Routine Cleaning and Sanitation

The enumeration of total mesophilic aerobes is one of the most common parameters used to assess the microbiological quality of food contact surfaces [22,23], whereas coliforms are generally used as a hygiene indicator. Coliforms include different genera, namely *Enterobacter*, *Klebsiella*, *Citrobacter*, *Serratia*, *Yersinia* and *Escherichia*. Within the latter genus, the species of faecal origin *E. coli* includes pathogenic (enteroinvasive or enterotossigenic) strains, thus explaining the generally required absence of this microorganism in processed foods and food environments.

To date, very few internationally accepted standards have been published to define acceptable levels of microbial contamination on surfaces (Annex of Commission Decision 2001/471/EC) [19], and no reference microbial limits are available for food contact surfaces at mass catering establishments. In this study, a threshold of 10 (for total mesophilic aerobes) and 1 (for coliforms and *E. coli*) cfu/cm^2^ was set for the definition of microbiological acceptability.

Table 2 reports the frequency of unacceptable samples based on these microbiological limits whereas Table 3 shows the results of ANOVA and minimum, maximum, and average viable counts of total mesophilic aerobes and coliforms. None of the samples analysed were positive for *E. coli*.

**Figure 2:**
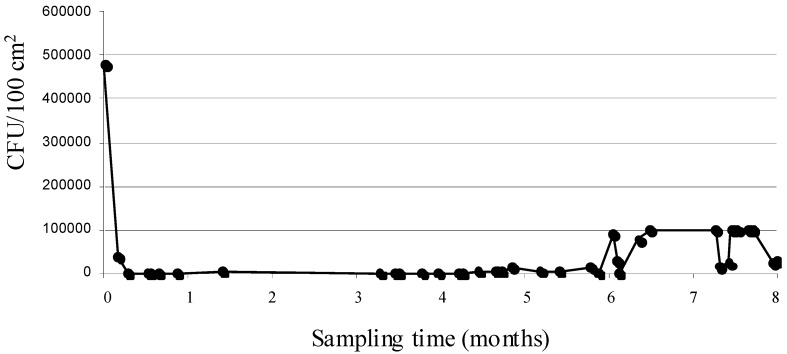
Eight-month total mesophilic aerobes counts trend for raw meat chopping board CFU Colony Forming Units.

Significantly higher percentages of unacceptable samples were found for the multipurpose (52.5%) and raw meat chopping-boards (47.5%), respectively. This finding is in quite good agreement with those relating to ATP bioluminescence measurements. In particular, for the raw meat chopping-board, an almost comparable trend to that of the mean RLU/100 cm^2^ was seen during the 8-month monitoring period (Figure 2), thus confirming the criticalities which emerged in a former study carried out at the same canteen [18]. Even the analysis of variance and HSD multiple comparisons (Table 3) showed that this CP was characterised by an overall significantly higher contamination than all the remaining surfaces, that did not differ among each other.

**Table 3:** ANOVA results (**a**) and multiple comparisons among mean values and range of data variation (minimum and maximum values) for each surfaces and variables analyzed (**b**) after routine cleaning and sanitation of food contact surfaces.

a.													
Sources of Variation	*df*	RLU		TMA		C							
Surfaces	9	***		***		***							
Time	39	ns		ns		ns							
Error	351												
**b.**													
**Surface**	**RLU/100 cm^2^**						**TMA cfu/100 cm^2^**			**Coliforms cfu/100 cm^2^**			**Ec** **cfu/100 cm^2^**
	**Min**		**Max**		Means *		**Min**	**Max**	**Means** *	**Min**	**Max**	**Means** *
Raw meat preparation table	18		1,568		183.05 ^b^		n.d.	1.1 × 10^4^	3.6 × 10^2^^b^	n.d.	n.d.	n.d.	n.d.
Raw meat chopping-board	41		451,593		59,548.05 ^a^		n.d.	4.8 × 10^5^	4.1 × 10^4^^a^	n.d.	1.8 × 10^2^	7.0 × 10 ^b^	n.d.
Raw meat knife	25		7,713		843.02 ^b^		n.d.	1.1 × 10^4^	9.7 × 10^2^^b^	n.d.	7.3 × 10^2^	2.0 × 10 ^b^	n.d.
Raw vegetable preparation table	35		12,695		811.37 ^b^		n.d.	8.2 × 10^3^	7.1 × 10^2^^b^	n.d.	3.0 × 10^2^	2.0 × 10 ^b^	n.d.
Raw vegetable knife	37		30,770		3,181.30 ^b^		n.d.	1.0 × 10^5^	5.9 × 10^3^^b^	n.d.	1.5 × 10^2^	1.0 × 10 ^b^	n.d.
Vegetable washer	11		3,041		332.45 ^b^		n.d.	2.3 × 10^4^	7.1 × 10^2^^b^	n.d.	n.d.	n.d.	n.d.
Multipurpose chopping-board	90		35,864		4,105.57 ^b^		n.d.	1.0 × 10^5^	8.1 × 10^3^^b^	n.d.	4.7 × 10^3^	2.2 × 10^2^^a^	n.d.
Cooked meat slicing machine	27		2,855		437.43 ^b^		n.d.	1.3 × 10^3^	1.3 × 10^2^^b^	n.d.	n.d.	n.d.	n.d.
Table for customers	25		4,243		526.32 ^b^		n.d.	2.2 × 10^2^	1.0 × 10 ^b^	n.d.	n.d.	n.d.	n.d.
Knife for customers	9		934		163.95 ^b^		n.d.	2.8 × 10^2^	1.0 × 10 ^b^	n.d.	n.d.	n.d.	n.d.

Notes: In panel **a**: df = degrees of freedom; ns = not significant; *** = significant at *p* < 0.001. In panel **b**: * For each variable means followed by different letters are significantly different (*p* < 0.05) RLU Relative Light Units; TMA Total Mesophilic Aerobes; Ec *Escherichia coli*; n.d. not detected (no colonies were grown on plates inoculated with 1 mL of undiluted sample).

The mean comparison for coliform contamination indicated that the multipurpose chopping-board was the most critical surface, thus confirming that the higher the number of types of food being processed, the higher is the potential for cross-contamination with this bacteria group.

### 3.4. Comparison between ATP Measurement and Viable Bacterial Cell Counting

Monitoring of ATP bioluminescence and viable counting of total mesophilic aerobes was comparatively evaluated. A noticeably higher number of unacceptable compared with acceptable samples was recorded by the ATP with respect to the microbiological technique (247 *versus* 76, respectively). In more detail, 146 samples that were evaluated as acceptable by ATP measurement were confirmed as acceptable by viable counting and 69 samples that were defined as unacceptable by ATP measurement were confirmed as unacceptable by the microbiological technique; on the other hand, 178 samples that resulted unacceptable using the ATP measurement were evaluated as acceptable by viable counting, whereas seven samples that were defined as unacceptable by viable counting resulted acceptable using the ATP measurement. A comparison of the monitoring of ATP bioluminescence and viable counting of coliforms showed a higher divergence in the ratio of unacceptable *versus* acceptable samples (namely 247 *vs.* 18 samples); in more detail 153 samples that were determined as acceptable by ATP measurement were confirmed acceptable by viable counting and 18 samples that were unacceptable by ATP measurement were confirmed unacceptable by viable counting; conversely, 229 samples that were unacceptable with the ATP measurement were acceptable with viable counting of coliforms.

In both cases, a high number of samples that were deemed “dirty” by ATP bioluminescence measurement proved to be not at risk in terms of either total mesophilic aerobes (215 samples) or coliform (171 samples) load, thus suggesting the high restrictiveness of the ATP benchmark values adopted. A study carried out by Carrascosa *et al.* [24] in dairy plant surfaces highlighted the low specificity of ATP-based technique for differentiating the remains of microbial organic content supporting the need to couple the measures of ATP to the microbial viable counts. Previous authors who used similar ATP systems for the monitoring of different surfaces at medical-surgical intensive care units suggested 500 and 250 RLU as an achievable and appropriate benchmark value, respectively [25,26]; however, in the first study, as literally stated by the authors “a very basic cleaning schedule was in existence”, thus implying that lower bioluminescence readings might have been consistently achieved through the implementation of a validated, well designed cleaning plan. If, on the one hand, the definition of too low benchmark values might lead to the execution of excessively scrupulous cleaning procedures, and hence to a waste of time, money and energy, on the other hand, the setting of too high benchmark values might lead to an underestimation of the risk associated with microbiological contamination, which is undoubtedly to be avoided. Analogously to what was suggested by Moore *et al.* [15] for hospital wards, cleaning CPs at food production plants or mass catering establishments should be treated as separate environments and different cleaning acceptability limits set accordingly.

A highly significant correlation (*r* = 0.99, *p* < 0.001) between the means of RLU and viable counts of total mesophilic aerobes confirmed the strong association of these two parameters with the level of surface contamination; both RLU and total mesophilic aerobes counts were not significantly correlated with coliform level (*r* = 0.40 and 0.28, respectively).

A reasonable correlation between microbial counts and ATP readings has previously been demonstrated on a number of surface samples from milk plants [27], poultry carcasses [28] and hands [29], although in other research studies, no correlation was found between the two parameters [15,30].

## 4. Conclusions

The overall results collected in this and some other studies using a similar analytical approach [15,21,30] clearly demonstrated how bioluminescence ATP monitoring cannot substitute the traditional quantification of microbial load on food contact surfaces. However, even in this study, this technology proved to have potential for the real time monitoring of surface cleanliness, for the verification of cleaning procedures within an HACCP plan, and for the implementation of corrective action against poor hygiene, such as the re-cleaning of unacceptable surfaces or the substitution/regeneration of worn work surfaces. In this regard, one of the major advantages of ATP bioluminescence monitoring lies in the self-evaluation by the staff responsible for the cleanliness and sanitation which derive from the correct execution of SSOP [31,32].

## References

[1] Newell D.G., Koopmans M., Verhoef L., Duizer E., Aidara-Kane A., Sprong H., Opsteegh M., Langelaar M., Threfall J., Scheutz F. (2010). Food-borne diseases—The challenges of 20 years ago still persist while new ones continue to emerge. Int. J. Food Microbiol..

[2] Petruzzelli A., Blasi G., Masini L., Calza L., Duranti A., Santarelli S., Fisichella S., Pezzotti G., Aquilanti L., Osimani A. (2010). Occurrence of *Listeria monocytogenes* in salami manufactured in the Marche Region (central Italy). J. Vet. Med. Sci..

[3] Petruzzelli A., Foglini M., Vetrano V., Paolini F., Orazietti N., Ambrosini B., Osimani A., Clementi F., Tavoletti S., Tonucci F. (2014). The occurrence of thermotolerant *Campylobacter* spp. in raw meat intended for public catering. Public Health.

[4] Gorman R., Bloomfield S., Adley C.C. (2002). A study of cross-contamination of food-borne pathogens in the domestic kitchen in the Republic of Ireland. Int. J. Food Microbiol..

[5] Osimani A., Aquilanti L., Tavoletti S., Clementi F. (2013). Evaluation of the HACCP system in a university canteen: Microbiological monitoring and internal auditing as verification tools. Int. J. Environ. Res. Public Health.

[6] Osimani A., Aquilanti L., Tavoletti S., Clementi F. (2013). Microbiological monitoring of air quality in a university canteen: An 11-year report. Environ. Monit. Assess..

[7] Martinon A., Cronin U.P., Quealy J., Stapleton A., Wilkinson M.G. (2012). Swab sample preparation and viable real-time PCR methodologies for the recovery of *Escherichia coli*, *Staphylococcus aureus* or *Listeria monocytogenes* from artificially contaminated food processing surfaces. Food Control.

[8] Shi X., Zhu X. (2009). Biofilm formation and food safety in food industries. Trends Food Sci. Technol..

[9] Simoes M., Simoes L.C., Vieira M.J. (2010). A review of current and emergent biofilm control strategies. LWT-Food Sci. Technol..

[10] Davey M.E., O’Toole G.A. (2000). Microbial biofilms: From ecology to molecular genetics. Microbiol. Mol. Biol. Rev..

[11] Srey S., Jahid I.K., Ha S.D. (2013). Biofilm formation in food industries: A food safety concern. Food Control.

[12] (2004). Regulation (EC) No. 852/2004 of the European Parliament and the Council of 29 April 2004 on the Hygiene of Foodstuffs. http://Eur-lex.europa.eu/LexUriServ/LexUriServ.do?uri=OJ:L:2004:139:0001:0054:en:PDF.

[13] Cenci-Goga B.T., Ortenzi R., Bartocci E., Codega de Oliveira A., Clementi F., Vizzani A. (2005). Effect of the implementation of HACCP on the microbiological quality of meals at a university restaurant. Foodborne Pathog. Dis..

[14] Petruzzelli A., Foglini M., Paolini F., Framboas M., Altissimi M.S., Haouet N.M., Mangili P., Osimani A., Clementi F., Cenci T. (2014). Evaluation of the quality of foods for special diets produced in a school catering facility within a HACCP-based approach: A case study. Int. J. Environ. Health Res..

[15] Moore G., Smyth D., Singleton J., Wilson P. (2010). The use of adenosine triphosphate bioluminescence to assess the efficacy of a modified cleaning program implemented within an intensive care setting. Amer. J. Infect. Control.

[16] Hawronskyj J.M., Holah J. (1997). ATP: A universal hygiene monitor. Trends Food Sci. Technol..

[17] Approval of Guidelines Regarding the Procedural Uniformity for the Release of the Health Card and for the Training of Staff in the Food Industry. http://www.salute2000.it/documenta_file/DGR_2173_2002.pdf.

[18] Osimani A., Babini V., Aquilanti L., Tavoletti S., Clementi F. (2011). An eight-year report on the implementation of HACCP in a university canteen: Impact on the microbiological quality of meals. Int. J. Environ. Health Res..

[19] Commission Decision of 8 June 2001 Laying down Rules for the Regular Checks on the General Hygiene Carried out by the Operators in Establishments According to Directive 64/433/EEC on Health Conditions for the Production and Marketing of Fresh Meat and Directive 71/118/EEC on Health Problems Affecting the Production and Placing on the Market of Fresh Poultry Meat. http://eur-lex.europa.eu/legal-content/EN/TXT/?uri=CELEX:32001D0471.

[20] Steele R.G.D., Torrie J.H. (1980). Principles and Procedures of Statistics—A Biometrical Approach.

[21] Aycicek H., Oguz U., Karci K. (2006). Comparison of results of ATP bioluminescence and traditional hygiene swabbing methods for the determination of surface cleanliness at a hospital kitchen. Int. J. Hyg. Environ. Health.

[22] Çetin Ö., Kahraman T., Kemal Büyükünal S. (2006). Microbiological evaluation of food contact surfaces at red meat processing plants in Istanbul, Turkey. Ital. J. Anim. Sci..

[23] Olgunoglu I.A. (2010). Determination of microbiological contamination sources of blue crabmeat (*Callinectes sapidus Rathbun*, 1896) during pasteurization process. Pak. J. Zool..

[24] Carrascosa C., Saavedra P., Millàn R., Jaber J.R., Pérez E., Grau R., Raposo A., Mauricio C., Sanjuàn E. (2012). Monitoring of cleanliness and disinfection in dairies: Comparison of traditional microbiological and ATP bioluminescence methods. Food Control.

[25] Griffith C.J., Cooper R.A., Gilmore J., Davies C., Lewis M. (2000). An evaluation of hospital cleaning regimes and standards. J. Hosp. Infect..

[26] Lewis T., Griffith C., Gallo M., Weinbren M. (2008). A modified ATP benchmark for evaluating the cleaning of some hospital environmental surfaces. J. Hosp. Infect..

[27] Murphy S.C., Kozlowski S.M., Bandler D.K., Boor K.J. (1998). Evaluation of ATP hygiene monitoring for trouble shooting fluid milk shelf life problems. J. Dairy Sci..

[28] Siragusa G.R., Dorsa W.J., Cutter C.N., Perino L.J. (1996). Use of a newly developed rapid microbial ATP bioluminescence assay to detect microbial contamination on poultry carcasses. J. Chemilum. Biolum..

[29] Marena C., Lodola L., Bulgheroni A., Carretto E., Zecca M., Maserati R., Zambianchi L. (2002). Assessment of handwashing practices using chemical and microbiological methods: Preliminary results from a prospective study. Amer. J. Infect. Control.

[30] Larson E.L., Aiello A., Gomez-Duarte C., Lin S.X., Lee L., Della-Latta P., Lindhardt C. (2003). Bioluminescence ATP monitoring as a surrogate marker for microbial load on hands and surfaces in the home. Food Microbiol..

[31] Amodio E., Dino C. (2014). Use of ATP bioluminescence for assessing the cleanliness of hospital surfaces: A review of the published literature (1990–2012). J. Infect. Public Health.

[32] Cooper R.A., Griffith C.J., Malik E.R., Obee P., Looker N. (2007). Monitoring the effectiveness of cleaning in four British hospitals. Amer. J. Infect. Control.

